# Potential value and limitations of different clinical scoring systems in the assessment of short- and long-term outcome following orthotopic liver transplantation

**DOI:** 10.1371/journal.pone.0214221

**Published:** 2019-03-21

**Authors:** Joerg Boecker, Zoltan Czigany, Jan Bednarsch, Iakovos Amygdalos, Franziska Meister, Daniel Antonio Morales Santana, Wen-Jia Liu, Pavel Strnad, Ulf Peter Neumann, Georg Lurje

**Affiliations:** 1 Department of Surgery and Transplantation, University Hospital RWTH Aachen, Aachen, Germany; 2 Department of Internal Medicine III, University Hospital RWTH Aachen, Aachen, Germany; 3 Department of Surgery, Maastricht University Medical Centre (MUMC), Maastricht, Netherland; University of Toledo, UNITED STATES

## Abstract

**Background:**

In an attempt to further improve liver allograft utilization and outcome in orthotopic liver transplantation (OLT), a variety of clinical scoring systems have been developed. Here we aimed to comparatively investigate the association of the Balance-of-Risk (BAR), Survival-Outcomes-Following-Liver-Transplant (SOFT), Preallocation-Survival-Outcomes-Following-Liver-Transplant (pSOFT), Donor-Risk-Index (DRI), and the Eurotransplant-Donor-Risk-Index (ET-DRI) scores with short- and long-term outcome following OLT.

**Methods:**

We included 338 consecutive patients, who underwent OLT in our institution between May 2010 and November 2017. For each prognostic model, the optimal cutoff values were determined with the help of the Youden-index and their diagnostic accuracy for 90-day post OLT-mortality and major postoperative complications was measured by the area under the receiver operating characteristic curve (AUROC). Patient- and graft survival were analyzed using the Kaplan-Meier method and the log-rank test. Morbidity was assessed using the Clavien-Dindo classification and the Comprehensive-Complication-Index.

**Results:**

BAR, SOFT, and pSOFT performed well above the conventional AUROC-threshold of 0.70 with good prediction of early mortality. Only BAR showed AUC>0.70 for both mortality and major morbidity. With the cutoffs of 14, 31, and 22 respectively for BAR, SOFT, and pSOFT, subgroup analysis showed significant differences (p<0.001) in morbidity and mortality, length of intensive care- and hospital-stay and early allograft dysfunction rates. Five-years patient survival was inferior in the high BAR, pSOFT, and SOFT groups.

**Conclusions:**

Out of all scores tested, the BAR-score had the best value in predicting both 90-day morbidity and mortality after OLT showing the highest AUCs. The pSOFT and SOFT scores demonstrated an acceptable accuracy in predicting 90-day morbidity and mortality. The used BAR, SOFT, and pSOFT cutoffs allowed the identification of patients at risk in terms of five-year patient survival. The DRI and ET-DRI scores have failed to predict recipient outcomes in the present setting.

## Introduction

Over the past 60 years, orthotopic liver transplantation (OLT) has evolved as the standard treatment for patients with end-stage liver disease and acute liver failure [1, 2]. While surgical techniques, organ preservation, intensive care management and immunosuppression have significantly improved during this time [3], the gap between supply and demand for liver allografts continues to increase. Several strategies, such as living donation, splitting of cadaveric grafts for two recipients and transplantation of extended criteria donor allografts (ECD) have been implemented to expand the donor pool [4–6].

To improve transparency and to promote fair allocation of allografts, the former center-based allocation policy was in 2006 replaced by the MELD allocation system (Model for End-Stage Liver Disease), that became mandatory for all participating centers within the Eurotransplant network. This “sickest-patient first policy" led to the current situation, where many recipients suffer from an advanced liver dysfunction and a poor general condition at the time of OLT [7]. In addition, more than 50% of potential donor allografts exhibit further risk factors, some of which include advanced donor age, expressed graft macrosteatosis and/or extended cold ischemic time (CIT) [8–10].

While the MELD-score is an accurate and well-documented 3-month mortality predictor for patients on the waiting list, it is not considered to be a suitable prediction tool for recipient outcomes following OLT [11, 12]. Therefore, instead of relying exclusively on the expertise and subjective assessment of the transplant surgeon, an objective, accurate and feasible prediction model of postoperative outcome ahead of the OLT procedure would facilitate liver allograft allocation to the most suitable recipients. To this aim, various clinical scoring systems, using donor and recipient factors, have been developed over the past 10–15 years [13–18].

The goal of our present study is to assess the performance of these scoring systems, i.e. (Balance-of-Risk (BAR), Survival-Outcomes-Following-Liver-Transplant (SOFT), Preallocation-Survival-Outcomes-Following-Liver-Transplant (pSOFT), Donor-Risk-Index (DRI), and the Eurotransplant-Donor-Risk-Index (ET-DRI)) in predicting short- and long-term outcome in patients underwent OLT at our institution.

## Patients and methods

### Study design and patients

Between May 2010 and November 2017, three-hundred and thirty-eight (n = 338) consecutive patients (age ≥ 18 years), who underwent OLT for end-stage liver disease at the University Hospital RWTH Aachen (UH-RWTH), were included in this study. Patients with study relevant missing data where calculation of one or more scores was not possible and living related transplantations were excluded (n = 10).

Laboratory MELD (labMELD) was used in all instances and exceptional MELD points were not considered. The study was conducted at the UH-RWTH in accordance with the requirements of the Institutional Review Board of the RWTH Aachen University (EK-047/18), the current version of the Declaration of Helsinki as well as the Declaration of Istanbul and the good clinical practice guidelines (ICH-GCP). Informed consent was waived due to the retrospective study design and collection of readily available clinical data. Recipient and donor characteristics are shown in Tables 1 and 2.

**Table 1:** Recipient characteristics.

**Recipient characteristics**	**(n or mean±SD)**	**(%)**
Gender (m/f)	221/107	67%/33%
Age in years	54±11	
BMI	27±5	
labMELD	20±11	
BAR-score^1^	9±6	
SOFT-score^2^	12±11	
pSOFT-score^2^	16±12	
pre-transplant life support^3^	40	12%
pre-transplant ICU	87	27%
pre-transplant abdominal surgery	128	39%
pre-transplant encephalopathy	127	39%
pre-transplant ascites	197	60%
**Indication for OLT**	**(n or mean±SD)**	**(%)**
Alcoholic cirrhosis	78	24%
HCC	75	23%
ALF	37	11%
PSC/PBC	33	10%
Viral (Hepatitis)	22	7%
Graft failure	22	7%
AIH	9	3%
Other	52	15%

m, male; f, female, SD, standard deviation; BMI, body mass index; labMELD, laboratory Model for End Stage Liver Disease; ICU, intensive care unit; OLT, orthotopic liver transplantation; HCC, hepatocellular carcinoma; ALF, acute liver failure; PSC, primary sclerosing cholangitis; PBC, primary biliary cholangitis; AIH, autoimmune hepatitis; BAR, Balance of Risk; pSOFT, preallocation Survival Outcomes Following Liver Transplant; SOFT, Survival Outcomes Following Liver Transplant

^1^Refers to Dutkowski et al.[17]

^2^Refers to Rana et al.[16]

^3^Life support is defined as dialysis and/or mechanical ventilation before transplantation

**Table 2:** Donor characteristics.

**Donor characteristics**	**(n or mean±SD)**	**(%)**
Gender (m/f)	174/154	53%/47%
Age in years	56±15	
BMI	29±7	
Extended criteria donor^1^	204	62%
DRI^2^	1.77±0.34	
ET-DRI^3^	1.93±1.68	
**Donor cause of death**	**(n or mean±SD)**	**(%)**
CVA	211	64%
Anoxia	65	20%
Trauma	41	13%
Other	11	3%
**Allocation type**	**(n or mean±SD)**	**(%)**
Local	16	4%
Regional	156	48%
National	156	48%

m, male; f, female, SD, standard deviation; BMI, body mass index; DRI, Donor Risk Index; ET-DRI, Eurotransplant-Donor Risk Index; CVA, Cerebrovascular accident

^1^Refers to German Medical Chamber Guidelines[19]

^2^Refers to Feng et al.[13]

^3^Refers to Braat et al.[23]

### Data collection and follow-up

Data were obtained from a prospectively maintained institutional database and analyzed retrospectively. Pre-transplant labMELD, DRI, ET-DRI, SOFT, pSOFT and BAR score were calculated as described below. Extended criteria donor allografts were defined according to the definition of the German Medical Chamber (donor age>65-years, ICU with mechanical ventilation>7 day, BMI>30, histologically confirmed graft steatosis>40%, serum sodium>165 mmol/L, serum alanine and/or aspartate amino-transferase>3x higher as the reference level, serum total bilirubine>2mg/dL) [19]. To assess post-transplant early allograft dysfunction (EAD) the Olthoff criteria were adopted [20]. Postoperative morbidity was evaluated for all surgical complications registered during the first 90-days following OLT according to the Clavien-Dindo classification (CD) and the Comprehensive Complication Index (CCI) [21, 22]. Postoperative transfusions were defined as blood products given within the first 7 days following OLT. Blood products administered later during the postoperative course and within the first 90-days were assessed among the postoperative complications. Length of ICU-stay represents the initial stay after the OLT-procedure until the transfer of the patient to a standard care transplantation ward. Readmission to ICU was assessed as part of the total hospital stay. Hospital stay was defined by the date of admission for OLT and the day of discharge from the UH-RWTH.

Each patient was assessed regularly by the referring hepatologist or the local outpatient clinic. The follow-up examinations included a clinical examination, standard blood test with follow-up tumor markers and cross-sectional imaging if applicable.

### Score models calculated

The analyzed prediction models (DRI, ET-DRI, SOFT, pSOFT and BAR) were calculated as described before [13, 16, 17, 23]. Further details on the calculation of the used scores are available as supporting information (S1 Appendix). Local allocation was defined as the procurement area of the UH-RWTH, whereas the rest of Germany was regarded as a regional allocation. The rest of the Eurotransplant region was considered as national or extra-regional sources, depending on the calculated prediction model [13, 14].

### Study endpoints and statistical analysis

Ninety-day mortality following OLT was chosen as the primary endpoint for the assessment of the predictive abilities of the various scores. As secondary endpoints, 90-day morbidity and 5-year graft- and patient survival were analyzed.

The discriminative ability of the various score models for the prediction of 90-day- survival and major complications (CD≥3b) was compared using the receiver operating characteristic (ROC) curve analysis calculating the area under the receiver operating characteristic curve (AUROC). The respective cutoff values of the potential prognostic models were selected with the help of the best Youden-index. The Hosmer-Lemeshow chi^2^ goodness-of-fit test was applied to test model suitability. For analysis of categorical data, the Chi-square test and the Fisher’s exact test, for comparison of continuous variables the Mann-Whitney U test were applied. The prognostic value of the various clinical scores was demonstrated in the subgroup comparisons using the odds-ratios and 95% confidence intervals from the univariable logistic regression analysis. The Spearman correlation coefficient was used to express further potential association between the assessed scores and various clinical outcome measures. To visualize patient and graft survival the Kaplan-Meier method was used. Survival data was analyzed with the log-rank test. All p-values<0.05 were considered statistically significant.

Statistical analysis was performed using SPSS Statistics v24 (IBM Corp., Armonk, NY, USA) and respective graphs were generated using Prism v7.0 (GraphPad Software, La Jolla, California, USA).

## Results

### Recipient and donor characteristics

After applying the above-mentioned inclusion- and exclusion criteria, 328 out of 338 consecutive OLTs were included in the analysis. The mean age of all the recipients was 54±11 years. Some 221 (67%) recipients were male and 107 (33%) were female. The mean pre-transplant laboratory MELD-score was 20±11. The most common indications for OLT were alcoholic cirrhosis (24% (78/328)) and hepatocellular carcinoma (23% (75/328)). Recipient characteristics and indications leading to listing for OLT are summarized in Table 1.

The mean donor age was 56±15 years. Some 174 (53%) donors were male and 154 (47%) were female. Mean donor BMI was 29±7. In compliance with the German law, all donors were donors after brain death (DBD), with cerebrovascular accidents (64% (211/328)) as the leading cause of death, followed by anoxia (20% (65/328)) and trauma (13% (41/328)). Sixty-two percent (62% (204/328)) of the transplanted livers fulfilled the ECD criteria. Further donor characteristics of interest are displayed in Table 2.

### Perioperative outcome

The mean CIT was 8.5±2 hours. Mean warm ischemic time (WIT) was 45±7 min (Table 3). Some 95% (307 out of 328) of all recipients developed post-transplant complications within the first 90-day following OLT according to the definitions of the Clavien-Dindo classification, however, a large part of these were minor complications (CD<3b). Major complications (CD ≥3b) were found in 174 cases (53%) [24]. The mean cumulative 90-day CCI for our OLT cohort was 55±32 (Table 3). Mean hospital stay was 41±36 days, mean ICU-stay was 15±25 days (Table 3). Early allograft dysfunction occurred in 94 cases (29%). Graft loss within the first 90 days occurred in 38 cases (12%). Twenty-two patients (7%) died within 90-days after receiving OLT (Clavien-Dindo classification 5).

**Table 3:** Perioperative outcomes.

Perioperative outcomes	(n or mean±SD)	(%)
CIT in minutes	507±124	
WIT in minutes	45±7	
Intraoperative PLT Units	1±2	
Intraoperative RBC Units	10±10	
Intraoperative FFP Units	18±11	
Postoperative PLT Units	1±2	
Postoperative RBC Units	4±7	
Postoperative FFP Units	7±12	
CD1^1^	15	5
CD2	66	20
CD3	113	35
CD4	91	28
CD5	22	7
Graft loss 90-day (including CD5)	38	12
Early allograft dysfunction (EAD)^2^	94	32
90-day CCI^3^	55±32	
ICU stay in days	15±25	
Hospital stay in days	41±36	

CIT, cold ischemic time; WIT, warm ischemic time; PLT unit, Platelet unit; RBC unit, Red blood cell unit; FFP unit, Fresh frozen plasma unit; CD, Clavien-Dindo; CCI, comprehensive complication index; SD, standard deviation; ICU, intensive care unit

^1^Refers to Dindo et al. [35]

^2^Refers to Olthoff et al.[20]

^3^Refers to Slankamenac et al. [21]

### Impact of the BAR, pSOFT, SOFT, DRI, and ET-DRI and their determined cutoffs on postoperative morbidity and mortality

The mean values and standard deviations for the different scores were DRI 1.77±0.34, ET-DRI 1.93±1.68, SOFT score 16±12, pSOFT score 12±11 and for BAR score 9±11 (Tables 1 and 2).

The areas under the receiver operating curve (AUROC) for the prediction of 90-day mortality were 0.847 for the BAR (CI 0.761–0.934; p<0.001), 0.837 for the SOFT (CI 0.736–0.939; p<0.001) and 0.821 for the pSOFT-scores (CI 0.714–0.928; p<0.001). The DRI and ET-DRI revealed AUROCs of 0.608 and 0.572 respectively. For the prediction of major complications (CD≥3b), AUROC for the BAR score was 0.709 (CI 0.654–0.765; p<0.001), for SOFT and pSOFT 0.680 and 0.661 (CI 0.623–0.738 and 0.602–0.720; each p<0.001) respectively. The DRI and ET-DRI showed a c-statistic<0.6 (0.535 and 0.555; p = 0.472 and p = 0.492, respectively) (Table 4). The goodness-of-fit testing, calculated for 90-day mortality and major morbidity, revealed a satisfactory model fit for each of the used scores (Table 4). The optimal score cutoff values were determined by the Youden-index and are shown in Table 5.

**Table 4:** AUROC analysis and goodness-of-fit testing for the various scores based on 90-day mortality and major complications (CD≥3b).

Score	AUC	SE	95% CI	p-value	chi^2^*	p-value^#^
**90-day mortality**						
BAR	0.847	0.044	0.761–0.934	**<0.001**	6.272	0.508
pSOFT	0.821	0.054	0.714–0.928	**<0.001**	12.946	0.114
SOFT	0.837	0.052	0.736–0.939	**<0.001**	6.056	0.641
DRI	0.608	0.058	0.493–0.722	0.099	2.211	0.974
ET-DRI	0.572	0.063	0.448–0.696	0.268	13.974	0.082
**Major complications (CD≥3b)**						
BAR	0.709	0.028	0.654–0.765	**<0.001**	8.718	0.274
pSOFT	0.661	0.030	0.602–0.720	**<0.001**	13.445	0.097
SOFT	0.680	0.030	0.623–0.738	**<0.001**	12.246	0.141
DRI	0.535	0.032	0.472–0.598	0.273	9.925	0.270
ET-DRI	0.555	0.032	0.492–0.618	0.088	10.796	0.214

*Hosmer-Lemeshow chi^2^

# in case of a p-value of <0.05 the test would reject the null hypothesis of an adequate fit. 95% CI: 95 Confidence Interval, AUC: Area under the curve; SE, standard error; BAR, Balance of Risk; pSOFT, preallocation Survival Outcomes Following Liver Transplant; SOFT, Survival Outcomes Following Liver Transplant; DRI, Donor Risk Index; ET-DRI, Eurotransplant-Donor Risk Index

**Table 5:** Specificity, sensitivity, and Youden indices to determine the best cutoff points for the BAR, pSOFT, SOFT, DRI, and ET-DRI scores based on 90-day mortality.

Score	Positive if >	Sensitivity	Specificity	Youden index
BAR	14	0.762	0.818	0.580
pSOFT	22	0.714	0.878	0.592
SOFT	31	0.714	0.924	0.638
DRI	1.75	0.762	0.498	0.260
ET-DRI	1.63	0.905	0.310	0.215

BAR, Balance of Risk; pSOFT, preallocation Survival Outcomes Following Liver Transplant; SOFT, Survival Outcomes Following Liver Transplant; DRI, Donor Risk Index; ET-DRI, Eurotransplant-Donor Risk Index

Next, we analyzed the ability of the different scores to stratify our patient cohort into high- and low-risk groups based on morbidity and mortality. As shown in Table 6, the subgroups of patients over the defined cutoff score values had significantly increased rates of major complications, CCI, 90-day mortality, and longer ICU- and hospital stay in case of the BAR-, pSOFT, and SOFT scores. Only the defined pSOFT cutoff was able to stratify patients concerning a higher incidence of EAD. In case of DRI and ET-DRI no significant differences were found (Table 6).

**Table 6:** Stratification of the postoperative outcome based on the determined BAR, pSOFT, SOFT, DRI and ET-DRI cutoffs.

			Odds-ratio (95% CI)	p-value^#^
**BAR-Score**	**>14(n = 72)**	**≤14(n = 256)**		
90-day CD3b&4 complications n (%)*	42	110	3.791 (1.970–7.295)	**<0.001**
**90-day CCI cutoff 60**	53	77	6.448 (3.580–11.613)	**<0.001**
**90-day mortality, CD5 n (%)**	16	6	11.857 (4.441–31.657)	**<0.001**
**Early allograft dysfunction n (%)**	23	71	1.235 (0.700–2.179)	0.466
**ICU stay (days)**	33±37	9±18	n.a.	**<0.001**^**§**^
**Hospital stay (days)**	67±49	34±28	n.a.	**<0.001**^**§**^
**pSOFT**	**>22(n = 53)**	**≤22(n = 275)**		
90-day CD3b&4 complications n (%)*	32	120	6.533 (2.644–16.145)	**<0.001**
**90-day CCI cutoff 60**	47	83	18.026 (7.418–43.804)	**<0.001**
**90-day mortality, CD5 n (%)**	15	7	15.056 (5.769–39.297)	**<0.001**
**Early allograft dysfunction n (%)**	22	72	2.047 (1.110–3.777)	**0.022**
**ICU stay (days)**	41±38	10±18	n.a.	**<0.001**^**§**^
**Hospital stay (days)**	76±51	34±28	n.a.	**<0.001**^**§**^
**SOFT**	**>31 (n = 39)**	**≤31 (n = 289)**		
90-day CD3b&4 complications n (%)*	22	130	12.777 (2.948–55.370)	**0.001**
**90-day CCI cutoff 60**	38	92	80.957 (10.946–598.776)	**<0.001**
**90-day mortality, CD5 n (%)**	15	7	25.089 (9.330–67.470)	**<0.001**
**Early allograft dysfunction n (%)**	15	79	1.717 (0.853–3.458)	0.130
**ICU stay (days)**	47±42	10±18	n.a.	**<0.001**^**§**^
**Hospital stay (days)**	73±38	37±34	n.a.	**<0.001**^**§**^
**DRI**	**>1.75 (n = 170)**	**≤1.75 (n = 158)**		
90-day CD3b&4 complications n (%)*	78	74	1.068 (0.682–1.673)	0.774
**90-day CCI cutoff 60**	73	36	1.320 (0.846–0.060)	0.221
**90-day mortality, CD5 n (%)**	16	6	2.615 (0.996–6.861)	0.051
**Early allograft dysfunction n (%)**	51	43	1.136 (0.702–1.837)	0.604
**ICU stay (days)**	16±27	13±24	n.a.	0.829^**§**^
**Hospital stay (days)**	44±42	38±29	n.a.	0.507^**§**^
**ET-DRI**	**>1.63 (n = 228)**	**≤1.63 (n = 100)**		
90-day CD3b&4 complications n (%)*	106	46	1.119 (0.690–1.814)	0.650
**90-day CCI cutoff 60**	91	39	1.022 (0.631–1.656)	0.930
**90-day mortality, CD5 n (%)**	19	3	2.909 (0.841–10.067)	0.092
**Early allograft dysfunction n (%)**	68	26	1.184 (0.697–2.014)	0.532
**ICU stay (days)**	14±25	15±27	n.a.	0.201^**§**^
**Hospital stay (days)**	41±38	41±32	n.a.	0.387^**§**^

*analysis was performed after the exclusion of patients with 90-day mortality, thus mortality could be reported separately

BAR, Balance of Risk; pSOFT, preallocation Survival Outcomes Following Liver Transplant; SOFT, Survival Outcomes Following Liver Transplant; DRI, Donor Risk Index; ET-DRI, Eurotransplant-Donor Risk Index; CD, Clavien-Dindo; CCI, comprehensive complication index; ICU, intensive care unit.

^#^Univariable logistic regression, except

^§^Mann-Whitney U-test

The association between perioperative outcome and the calculated values of the various scores were assessed further using the Spearman`s correlation coefficient. A moderately strong but significant positive association was observed between the BAR, pSOFT and SOFT score values and the days spent on ICU (BAR: r = 0.523 p<0.001; pSOFT: r = 0.511 p<0.001; SOFT: r = 0.502 p<0.001), length of hospital stay (BAR: r = 0.487 p<0.001; pSOFT: r = 0.534 p<0.001; SOFT: r = 0.532 p<0.001) as well as the cumulative 90-day CCI (BAR: r = 0.469 p<0.001; pSOFT: r = 0.434 p<0.001; SOFT: r = 0.441 p<0.001) (Table 7). No meaningful correlation was found between the above-mentioned outcome measures and the DRI and ET-DRI scores (Table 7).

**Table 7:** Association between the BAR, pSOFT, SOFT, DRI and ET-DRI scores and perioperative outcome parameters as well as pre-transplant labMELD.

	ICU Stay		Hospital stay		90-day CCI		labMELD	
	r	p	r	p	r	p	r	p
**BAR**	0.523	**<0.001**	0.487	**<0.001**	0.469	**<0.001**	0.879	**<0.001**
**pSOFT**	0.511	**<0.001**	0.534	**<0.001**	0.434	**<0.001**	0.723	**<0.001**
**SOFT**	0.502	**<0.001**	0.532	**<0.001**	0.441	**<0.001**	0.700	**<0.001**
**DRI**	0.025	0.657	0.049	0.377	0.047	0.395	0.030	0.587
**ET-DRI**	-0.032	0.566	-0.016	0.775	0.047	0.401	-0.021	0.706

ICU, intensive care unit; CCI, comprehensive complication index; r, Spearman correlation coefficient; p, p—value; BAR, Balance of Risk; pSOFT, preallocation Survival Outcomes Following Liver Transplant; SOFT, Survival Outcomes Following Liver Transplant; DRI, Donor Risk Index; ET-DRI, Eurotransplant-Donor Risk Index; labMELD, laboratory Model for End Stage Liver Disease

### Association of the BAR, pSOFT, SOFT, DRI, and ET-DRI scores with long-term graft- and patient survival

While the major focus of our study was on short-term outcomes, we also performed Kaplan-Meier curve analyses to indicate differences in long-term (5-year) survival. The 5-year cumulative patient survival rate was 76% for BAR≤14 vs. 69% for BAR>14 (p = 0.042), 77% for pSOFT≤22 vs. 57% for pSOFT>22 (p<0.001), 77% for SOFT≤31 vs. 50% for SOFT>31 (p<0.001), 81% for DRI≤1.75 vs. 69% for DRI>1.75 (p = 0.068) and 81% for ET-DRI≤1.63, 72% for ET-DRI>1.63 (p = 0.250).

**Fig 1:**
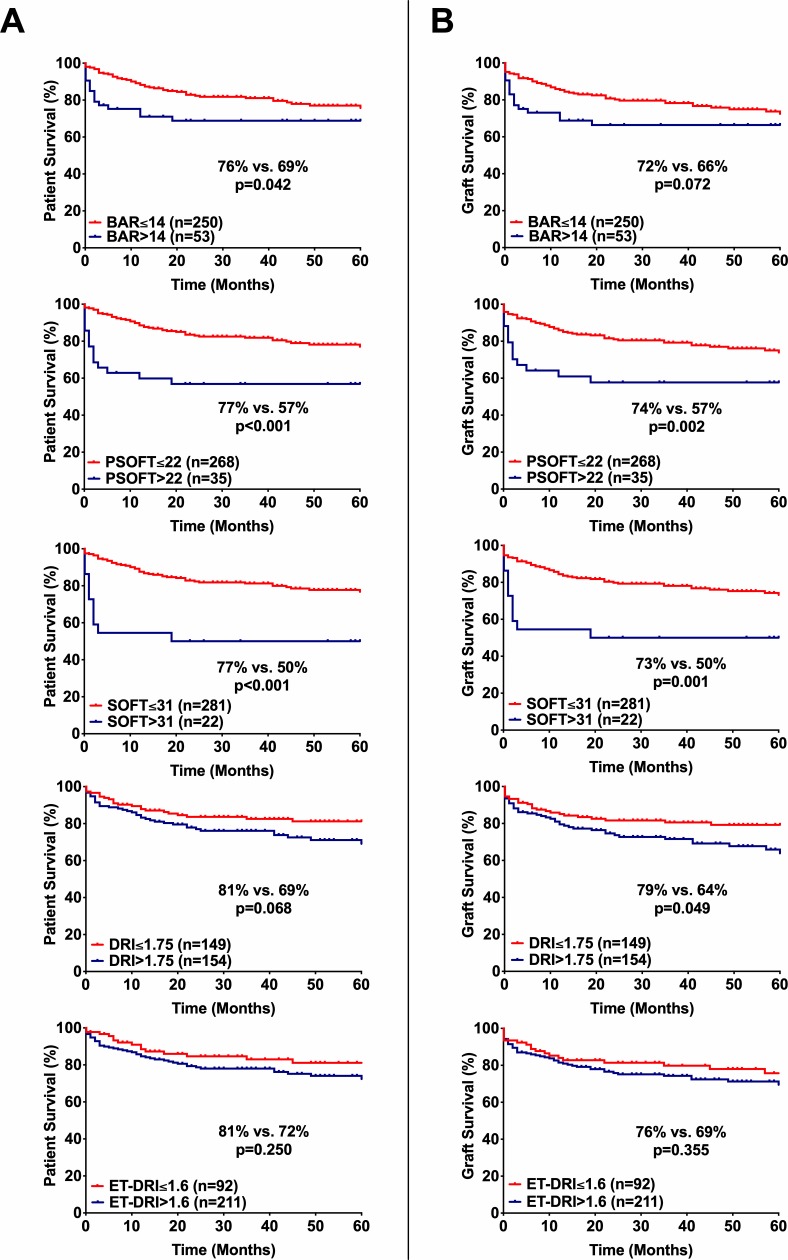
Patient- and graft survival stratified by the determined BAR-, pSOFT-, SOFT-, DRI- and ET-DRI-cutoff values. **(A)** Five-year patient survival according to the used score models. **(B)** Five-year graft survival according to the used score models. BAR, Balance of Risk; pSOFT, preallocation Survival Outcomes Following Liver Transplant; SOFT, Survival Outcomes Following Liver Transplant; DRI, Donor Risk Index; ET-DRI, Eurotransplant-Donor Risk Index.

Graft survival was 72% for BAR≤14 vs. 66% for BAR>14 (p = 0.072), 74% for pSOFT≤22 vs. 57% for pSOFT>22 (p = 0.002) and 73% for SOFT≤31 vs. 50% for SOFT>31 (p = 0.001), 79% for DRI≤1.75 vs. 64% for DRI>1.75 (p = 0.049) and 76% for ET-DRI≤1.63 vs. 69% for ET-DRI>1.63 (p = 0.355) (Fig 1).

## Discussion

An effective utilization of the existing organ donor pool with an optimal graft and recipient matching are of utmost clinical importance in solid organ transplantation and are currently primarily based on subjective clinical evaluation of the transplant surgeon. An objective risk-assessment tool that is able to reliably predict post-OLT outcomes is urgently needed to establish an objective and a more transparent allocation. Even though several prediction tools have been developed, none of them has found its way into the clinical routine yet. Based on this, we aimed to comparatively assess the predictive value of five differential clinical prediction tools (BAR, pSOFT, SOFT, DRI, ET-DRI) in the context of 90-day mortality/morbidity and 5-year graft- and patient survival in adult recipients of OLT.

Following its initial development, subsequent studies validated the DRI as a potential independent predictor of allograft failure in different MELD categories in the post-MELD era [8, 24, 25]. The DRI, which was formulated in the pre-MELD era in 2005, showed a c-statistic ranging from 0.500 to 0.650 in separate studies suggesting an already rather low association with outcome [13, 26]. Our own findings showed a comparably low AUROC of 0.608 for the prediction of 90-day mortality. These findings are likely attributed to the well-known shortcomings of the DRI such as the validation in the pre-MELD era, and the disregard of relevant recipient risk factors. Accordingly, in a survey performed by Mataya et al. on the value of the DRI in clinical decision making, 73% of the respondents believed that the DRI is not a feasible tool to predict morbidity and graft failure following OLT. Moreover, 88% even stated that there are misleading aspects accompanied with the index, such as its poor predictive ability, inclusion of irrelevant factors (e.g. ethnicity) and the omission of relevant factors (e.g. recipient factors and further important donor factors such as graft steatosis or vasopressor support) [27]. In the recent years, the DRI was adapted to the Eurotransplant setting by Braat et al., replacing the risk factors “ethnicity” and “height” with the parameters “latest GGT” und “rescue offer”. Braat et al. claimed that the ET-DRI may be a useful tool for liver allocation in the future [23]. However, with a c-statistic of 0.624 (overall graft survival), it appears to be a predictor of only limited utility. Later studies claimed an even lower AUROC of 0.480–0.520 [28]. While Schoening et al. found a significant value of ET-DRI for specific subgroups [29], our own findings showed a disappointing AUROC of 0.572 for 90-day mortality with ET-DRI presenting a limited impact in the prediction of early outcome following OLT in our cohort. This is in line with a study of Reichert et al., who found an AUROC of 0.477 for three months mortality and 0.524 for three months graft survival in their European cohort [30]. Overall the DRI and ET-DRI performed well below the conventional AUROC threshold of 0.700 in this as well as in previous studies, therefore these cannot be considered as suitable tools to predict morbidity and mortality after liver transplantation at this time.

In accordance with the c-statistic reported initially by Rana et al. (AUROC 0.7) [15, 16], our analysis showed a promising AUROC of 0.837 for the SOFT-score for the prediction of 90-day post-transplant mortality. The pSOFT-score, which utilizes 14 recipient risk factors and was developed to weigh the expected mortality risk prior to transplant versus the risk without transplantation, showed an AUROC of 0.821 in our cohort. Since an AUROC between 0.8 and 0.9 represents an excellent diagnostic accuracy, it seems that the SOFT- and the pSOFT-scores are suitable and attractive tools to predict 90-day mortality. Nevertheless, in case of the SOFT/pSOFT scores, the inclusion of multiple variables, some of them being partially subjective and only semi-quantitative (e.g. encephalopathy, ascites), and a complex statistical modeling impair the practical applicability for prompt clinical assessment and decision-making prior transplantation. With an AUROC of 0.680 and 0.661, respectively, SOFT and pSOFT displayed a limited value for the prediction of 3-month major morbidity. This finding is in accordance with Schlegel et al. who found a c-statistic of 0.605 for the prediction of 3-month morbidity (CD>3a) for the SOFT score in a selected population of high MELD-recipients (MELD score≥30) [18].

The BAR score constitutes a promising novel tool developed by Dutkowski et al. which evaluates not only donor- but also easily accessible recipient risk factors. In our cohort, the BAR score was the only measure to predict 90-day morbidity with a reasonable accuracy (AUROC>0.7), hence it seems to be suitable to stratify patients based on both 90-day mortality and morbidity. While other authors found AUROCs below 0.7 for the prediction on 90-day mortality using the BAR-score [31, 32], in the present study ROC analysis revealed a convincing AUROC of 0.847 for the prediction of 90-day mortality and a solid AUROC of 0.709 for major morbidity. This is in line with the findings of Schlegel et al. who reported a related c-statistic of 0.754 for severe complications (CD≥3b) and 0.734 for 90-day mortality in case of the BAR-score [18]. The robust nature of the BAR score in predicting outcomes has been confirmed in other cohorts including pediatric/adolescent patients as well as in recipients of living donor liver transplantation [33, 34].

In our subsequent analysis, the subgroups of patients with high BAR, pSOFT, and SOFT scores performed significantly worse in terms of almost all assessed short-term outcome measures including major complications, cumulative CCI scores, 90-day mortality, and the length of ICU- and hospital stay. Seemingly pSOFT demonstrated a potential benefit over the BAR and SOFT scores in stratifying patients at risk based on the incidence of EAD, however, further studies are needed to confirm this finding. As expected from the general performance of the DRI and ET-DRI scores in the ROC-analysis, the used DRI and ET-DRI cutoffs failed to stratify the patients into a low- and high-risk groups with regards to 90-day morbidity and mortality.

The association between early morbidity and mortality and the BAR-, SOFT-, and pSOFT-scores were further supported by their significant correlation with the length of ICU-stay, days of in-hospital care and 90-day CCI values. None of these factors showed a significant association neither with the DRI nor with the ET-DRI scores.

Although the used cutoff values for the BAR-, pSOFT-, SOFT-, DRI-, and ET-DRI-scores were not optimized for long-term survival, the clinical value of the BAR-, pSOFT, and SOFT scores was strengthened by their significant association with 5-year patient- (and graft) survival (Fig 1).

Of note, all five investigated scores have been criticized for lacking certain well-recognized donor risk factors such as the presence and severity of graft steatosis. The lack of clear guidelines on a standardized biopsy-harvesting approach within Eurotransplant and the non-standardized semi-quantitative pathological assessment of steatosis (macro- versus microvesicular steatosis) constitute significant barriers in the incorporation of this important risk factor into prognostic OLT models. Moreover, other important risk factors such as CIT are sometimes difficult to estimate [13].

The interpretation of our findings is certainly limited by the sample size and the retrospective nature of our single-center assessment. Notwithstanding these limitations, this report is one of the first comprehensive studies assessing and comparing the value and limitations of five different clinical outcome scoring systems for OLT, demonstrating the potential value of the BAR-, SOFT-, and pSOFT scores and an inferior performance of the DRI and ET-DRI scores. It should be noted that in the present study only the BAR score was able to predict 90-day major morbidity *and* mortality with a high accuracy (AUROC>0.700). Based on its excellent value in predicting both 90-day mortality and major morbidity and the very easy and feasible calculation, the BAR-score might become a useful tool in the German allocation system to predict postoperative outcomes. Based on the promising results observed with the SOFT/pSOFT scores as well, future studies should evaluate the clinical feasibility of these complex scores and any potential benefits compared to the BAR score in various patient cohorts using patient- and graft survival as primary endpoints. Despite these encouraging results, validation in a prospective multicenter setting is warranted before implementing any of these prognostic tools into the routine clinical practice.

## Supporting information

S1 Appendix Exact methods used to calculate DRI, ET-DRI, SOFT, P-SOFT, and BAR Scores. (PDF)
