# Supplementary material for: Potential value and limitations of different clinical scoring systems in the assessment of short- and long-term outcome following orthotopic liver transplantation
Source: PLoS One. 2019 Mar 21;14(3):e0214221. doi: 10.1371/journal.pone.0214221 (PMC6428268; doi:10.1371/journal.pone.0214221)
Supplement: S1 Appendix — (PDF) [file pone.0214221.s001.pdf]

S1 Appendix - Exact methods used to calculate DRI, ET-DRI, SOFT, P-SOFT, and BAR Scores

DRI:

The DRI includes eight donor and allograft factors: Age, cause of death (COD), race, donor type (Donation after cardiac death (DCD) versus donation after brain death (DBD)), height, partial/split allograft, allograft location (local/regional/national) and cold ischemia time (CIT). The DRI was calculated as described before [1]:

DRI = exp[(0.154 if 40≤age<50) + (0.274 if 50≤ age<60) + (0.424 if 60≤age<70) + (0.501 if 70≤age) + (0.079 if COD=anoxia) + (0.145 if COD=CVA) + (0.184 if COD=other) + (0.176 if race=African American) + (0.126 if race=other) + (0.411 if DCD) + (0.422 if partial/split) + (0.066 ((170–height)/10)) + (0.105 if regional share) + (0.244 if national share) + (0.010×cold time)]

ET-DRI:

The ET-DRI also includes eight donor and allograft factors: Age, COD, donor type (DCD vs. DBD), partial/split allograft, allograft location (local/regional/extraregional), CIT, gamma glutamyl transpeptidase (GGT) and rescue allocation. The ET-DRI was calculated as described before [2]:

ET-DRI = exp[0.960((0.154 if 40≤age<50) + (0.274 if 50≤age<60)+(0.424 if 60≤age <70)+(0.501 if 70≤age)+(0.079 if COD=anoxia) + (0.145 9 if COD=cerebrovascular accident) + (0.184 if COD=other) + (0.411 if DCD) + (0.422 if partial/split) + (0.105 if regional share) + (0.244 if national share)) + (0.010 9 (cold ischemia time 8h)) + 0.06((latest lab GGt (U/L) 50)/100) + (0.180 if rescue offer)].

SOFT and pSOFT:

The SOFT and pSOFT-score consist of 18 recipient and donor/allograft risk factors and 14 recipient risk factors, respectively. Each positive factor results in allotted score points (one positive point for every 10% increase in risk for death at 3 months, one negative point for every 10% decrease in risk for death at 3 months) which are added up to the final score (Range: 0-72). Recipient pretransplant factors: Age (0-4 points), BMI >35 (0-2 points), one or two previous transplants (0-9-14 points), previous abdominal surgery (0-2 points), albumin level >2g/dl (0-2 points), dialysis prior to transplantation (0-3 points), treated at ICU (0-6 points), hospital admission (0-3 points), labMELD score>30 (0-4 points), life support (ventilation) (0-9 points), encephalopathy (0-2 points), portal vein thrombosis (0-5 points), ascites (0-3 points) and portal bleed 48h (0-6 points). Donor factors: Age (-2-0-3 points), cause of death (0-2 points), creatinine level >1,5 mg/dL (0-2 points), national allocation (0-2 points) and CIT 0-6 h (-3 points) [3].

BAR:

The BAR score consists of two donor/allograft and four recipient factors. Each factor results in allotted score points which are added up to the final score (Range: 0-27): Donor age (0-1 point), CIT (0-1-2 points), recipient age (0-1-3 points), recipient MELD-Score (0-5-10-14 points), recipient life support pretransplant (0-3 points) and retransplantation (0-4 points) [4].

REFERENCES

1. Feng S, Goodrich NP, Bragg-Gresham JL, Dykstra DM, Punch JD, DebRoy MA, et al. Characteristics associated with liver graft failure: the concept of a donor risk index. American journal of transplantation : official journal of the American Society of Transplantation and the American Society of Transplant Surgeons. 2006;6(4):783-90. Epub 2006/03/17. doi: 10.1111/j.1600-6143.2006.01242.x. PubMed PMID: 16539636.

2. Braat AE, Blok JJ, Putter H, Adam R, Burroughs AK, Rahmel AO, et al. The Eurotransplant donor risk index in liver transplantation: ET-DRI. American journal of transplantation : official journal of the American Society of Transplantation and the American Society of Transplant Surgeons. 2012;12(10):2789-96. doi: 10.1111/j.1600-6143.2012.04195.x. PubMed PMID: 22823098.

3. Rana A, Hardy MA, Halazun KJ, Woodland DC, Ratner LE, Samstein B, et al. Survival outcomes following liver transplantation (SOFT) score: a novel method to predict patient survival following liver transplantation. American journal of transplantation : official journal of the American Society of Transplantation and the American Society of Transplant Surgeons. 2008;8(12):2537-46. Epub 2008/10/24. doi: 10.1111/j.1600-6143.2008.02400.x. PubMed PMID: 18945283.

4. Dutkowski P, Oberkofler CE, Slankamenac K, Puhan MA, Schadde E, Mullhaupt B, et al. Are there better guidelines for allocation in liver transplantation? A novel score targeting justice and utility in the model for end-stage liver disease era. Annals of surgery. 2011;254(5):745-53; discussion 53. Epub 2011/11/02. doi: 10.1097/SLA.0b013e3182365081. PubMed PMID: 22042468.
